# Short Eggshell Membrane Nanofibers–Chitosan Hydrogel with Dual-Functional Hemostasis and Shape Memory for Non-Compressible Wounds

**DOI:** 10.3390/gels12040324

**Published:** 2026-04-10

**Authors:** Shuang Zhao, Wei Jiang, Yating Gou, Shurui Zhu, Yutong Yuan, Biyun Li, Huihua Yuan

**Affiliations:** School of Life Sciences, Nantong University, Nantong 226019, China

**Keywords:** deep hemostasis, eggshell membrane, chitosan, hydrogel, shape memory

## Abstract

Effective hemostasis in deep and irregular wounds remains a critical clinical challenge. To address this, we developed a bioresorbable chitosan composite hydrogel reinforced with short eggshell membrane (ESM) nanofibers, which were obtained through cryogenic grinding. The resulting ESM/CCS hydrogel, crosslinked with citric acid, exhibited significantly enhanced properties compared to pure CCS hydrogel, including a 63% increase in mechanical strength, a two-fold improvement in shape memory, a 25.31% reduction in hemolysis, over 2% higher cytocompatibility, and more than 48% greater hemostatic efficiency. Structural characterization confirmed the successful integration of bioactive chitosan with collagen mimetic ESM nanofibers. This biomimetic approach synergistically combines mechanical reinforcement with biological functionality, highlighting its strong potential as an advanced hemostatic dressing for complex wound management.

## 1. Introduction

Uncontrolled hemorrhage remains a leading cause of preventable death in both traumatic injuries and surgical procedures [1], with deep and irregularly shaped non-compressible wounds presenting a particularly formidable clinical challenge [2]. In such complex wound geometries, conventional hemostatic techniques—including gauze packing and compression bandages—often prove inadequate due to their inability to conform to the wound bed and achieve effective sealing [3]. Although rapid hemorrhage control is critical for improving patient survival outcomes, currently available commercial dressings remain largely reliant on passive physical compression and absorption, offering limited efficacy in actively bleeding, geometrically complicated wounds [4]. This persistent therapeutic gap has motivated intensive research efforts toward the development of next-generation hemostatic materials that simultaneously integrate rapid pro-coagulant activity, excellent biocompatibility, and controlled biodegradability [5].

Among the diverse platforms explored, hydrogel-based dressings have emerged as particularly attractive candidates owing to their unique physicochemical properties at the wound interface [6]. Within the spectrum of hydrogel-forming polymers, chitosan—a naturally derived cationic polysaccharide obtained from chitin—has attracted substantial interest due to its inherent bioactivities. Chitosan exhibits intrinsic antimicrobial properties, excellent biocompatibility, biodegradability, and most importantly, hemostatic activity mediated through electrostatic interactions with negatively charged erythrocyte membranes and platelet activation [7]. However, a critical limitation persists: chitosan requires acidic solvents for dissolution and hydrogel formation, conditions that frequently compromise the structural integrity and mechanical performance of the resulting hydrogels [8]. This mechanical inadequacy—manifesting as insufficient strength, poor elasticity, and limited shape recovery—has substantially hindered the clinical translation of pure chitosan hydrogels for applications requiring robust mechanical performance.

To address these limitations, various reinforcement strategies have been systematically investigated. These approaches can be broadly categorized into: (1) polymeric blending with mechanically robust biopolymers such as crystalline cellulose or silk fibroin [9], (2) dynamic physical crosslinking incorporating bioactive molecules or nanoparticles to create hybrid networks [10], and (3) chemical crosslinking using naturally derived agents such as citric acid, which offers a non-cytotoxic alternative to synthetic crosslinkers like glutaraldehyde while simultaneously contributing to network stabilization [11]. Among these strategies, nanofiber reinforcement has emerged as a particularly promising approach due to its dual functionality [12]. Nanofibers not only provide mechanical reinforcement through load transfer and crack bridging mechanisms but also structurally mimic the fibrous architecture of native extracellular matrix (ECM) [13], potentially creating a more biomimetic microenvironment that supports cellular responses while improving mechanical properties [14]. However, conventional continuous nanofibers—typically fabricated through electrospinning—face significant challenges related to dispersion within hydrogel matrices. Their high aspect ratio and tendency to entangle result in heterogeneous distribution and poor interfacial integration, ultimately limiting their reinforcement efficiency [15]. In contrast, short nanofibrils offer substantially improved dispersibility, higher available surface area for polymer–fibril interactions, and more uniform network integration, positioning them as ideal candidates for hydrogel nanocomposite reinforcement [16].

Eggshell membrane (ESM), an abundant biological byproduct of the food industry, represents an exceptionally attractive yet underexploited source of natural nanofibrils [17]. Chemically, ESM is primarily composed of type I collagen [18], fibronectin, laminin, and various glycoproteins—protein components that bear striking structural and biochemical resemblance to human dermal ECM; ESM structurally and biochemically resembles human dermal proteins [19]. Morphologically, ESM exhibits a hierarchical fiber architecture comprising both outer and inner membrane layers, with fiber diameters ranging from 0.1 to 3 μm and interconnected porous networks that provide both mechanical integrity and biological functionality [20]. Importantly, ESM-derived materials have demonstrated inherent wound-healing properties, including promotion of cell adhesion, proliferation, and migration, attributable to the preserved bioactivity of its constituent ECM proteins [21]. With global egg production exceeding 80 million tons annually [22], ESM represents an abundantly available, eco-friendly, and cost-effective resource for biomedical innovation, simultaneously addressing the dual imperatives of waste valorization and advanced biomaterial development [23].

In this study, we report the development of a novel hemostatic hydrogel through the integration of ESM-derived nanofibrils into a citric-acid-crosslinked chitosan (CCS) matrix. To our knowledge, this specific combination—synergizing three functionally complementary components—has not been previously described. The design rationale uniquely capitalizes on: (1) the intrinsic hemostatic and antimicrobial bioactivity of chitosan; (2) the mechanical reinforcement capacity and collagen mimetic ECM architecture of ESM nanofibrils; and (3) the non-cytotoxic network stabilization provided by citric-acid-mediated crosslinking. We hypothesized that the superior dispersibility of ESM nanofibrils would enable homogeneous reinforcement throughout the chitosan network, while the biochemical similarity of ESM to native ECM proteins would impart additional biofunctional properties beyond mere mechanical enhancement. The resulting ESM/CCS composite hydrogel was systematically characterized for its structural, mechanical, and biological properties. Compared to pure CCS controls, the optimized composite demonstrated substantially enhanced mechanical strength, markedly improved shape memory capability enabling recovery after deformation, significantly reduced hemolytic activity, and superior hemostatic efficiency in both in vitro and in vivo models, while maintaining excellent cytocompatibility. By transforming waste-derived biological nanofibrils into a functional structural and bioactive component, this work establishes a new biomimetic paradigm for engineering advanced hemostatic dressings capable of addressing the previously unmet needs of complex, non-compressible wound management.

## 2. Results and Discussion

### 2.1. Preparation and Characterization of ESM

#### 2.1.1. Morphology of ESM

**Figure 1:**
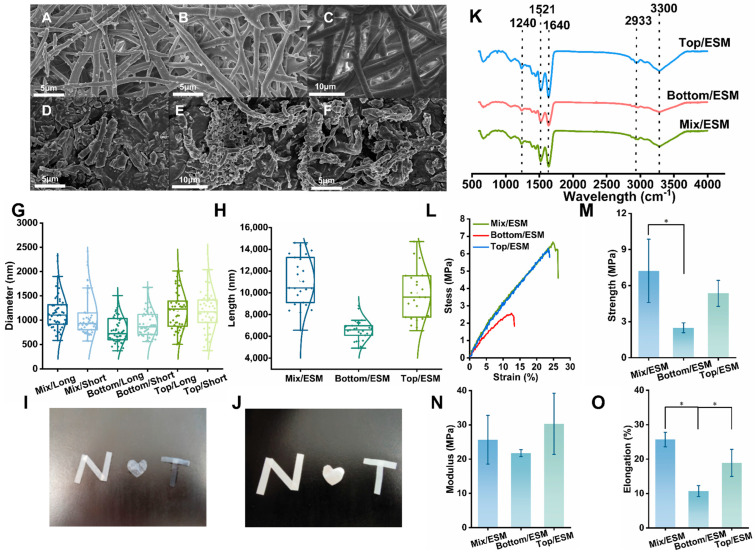
The characterization of ESM. (**A**–**C**) SEM images of the Mix/ESM, Bottom/ESM, Top/ESM; (**D**–**F**) SEM images of the he short fibers of Mix/ESM, Bottom/ESM, and Top/ESM; (**G**) Different fiber diameters (*n* = 50); (**H**) Different fiber lengths (*n* = 50); (**I**) Morphology of Mix/ESM, Top/ESM, and Bottom/ESM in wet environment; (**J**) Morphology of Mix/ESM, Top/ESM, and Bottom/ESM in dry environment; (**K**) Infrared spectra of Mix/ESM, Bottom/ESM, and Top/ESM; (**L**) The stress–strain curves of the Mix/ESM, Bottom/ESM, and Top/ESM; (**M**) The tensile strength of the Mix/ESM, Bottom/ESM, and Top/ESM. * *p* < 0.05 (*n* = 3); (**N**) Young’s moduli of the Mix/ESM, Bottom/ESM, and Top/ESM (*n* = 3); (**O**) The elongation rate of the Mix/ESM, Bottom/ESM, and Top/ESM. * *p* < 0.05 (*n* = 3).

Figure 1I and Figure 1J show the morphological differences in the membranes under wet and dry conditions, respectively. Figure 1I displays the membrane morphology in a wet environment, while Figure 1J presents the corresponding morphology in a dry state, revealing the structural stability under different environmental conditions. The surface morphologies of Mix/ESM, Bottom/ESM, Top/ESM, and their corresponding short fibers were characterized using scanning electron microscopy (SEM). As expected, all three native ESM variants exhibited highly cross-linked fibrous protein network structures, consistent with their biological role as structural membranes [24]. Notably, Mix/ESM displayed a relatively uniform pore distribution and fiber arrangement (Figure 1A), whereas Top/ESM presented a looser architecture with larger pores compared to Bottom/ESM, which featured densely cross-linked fibers and smaller pore sizes (Figure 1B,C). Quantitative analysis of fiber diameters using ImageJ software (1.54j) revealed average diameters of 1165 ± 370 nm for Mix/ESM, 798 ± 268 nm for Bottom/ESM (inner shell membrane), and 1196 ± 388 nm for Top/ESM (outer shell membrane) (Figure 1G). These measurements are in good agreement with previous reports [25], confirming that inner shell membrane fibers typically measure ≤2 μm in diameter and are approximately half the thickness of outer shell membrane fibers, which are known for their greater abundance, increased thickness, and higher surface roughness. Following cryogenic grinding, the resulting short fibers largely retained the morphological characteristics of their parent materials. Mix/ESM short fibers maintained a uniform distribution with moderate fiber thickness and pore size (Figure 1D). Bottom/ESM short fibers exhibited a more compact architecture, with finer fibers (924 ± 266 nm) and smaller pores, reflecting their inherently dense protein cross-linking (Figure 1E). Top/ESM short fibers, in contrast, appeared looser, with thicker fibers (1179 ± 425 nm) and larger pores, consistent with their less densely cross-linked native network (Figure 1F). Critically, the grinding process preserved fiber diameters while effectively reducing fiber lengths to approximately 10 μm—a key requirement for achieving homogeneous dispersion within composite matrices. Quantitative measurements confirmed diameters of 1077 ± 440 nm, 924 ± 266 nm, and 1179 ± 425 nm, and lengths of 11,117 ± 2303 nm, 6570 ± 947 nm, and 9982 ± 2422 nm for Mix/, Bottom/, and Top/ESM short fibers, respectively (Figure 1G,H). This outcome highlights two key advantages of the adopted processing strategy: (1) the method avoided damage to fiber diameter, thereby preserving the mechanical integrity essential for reinforcement; and (2) the process yielded uniformly dispersible short fibers with aspect ratios suitable for effective load transfer within hydrogel networks. Notably, among the three variants, Mix/ESM short fibers demonstrated the most uniform dimensional distribution, combining moderate fiber thickness with consistent length. This structural uniformity, together with their moderately dense cross-linking, suggests superior compatibility for composite applications where homogeneous dispersion and mechanical reinforcement are critical. Collectively, these findings establish Mix/ESM short fibers as a particularly promising candidate for the development of tailored hydrogel reinforcement systems.

#### 2.1.2. FTIR Analysis of ESM

Fourier transform infrared (FTIR) spectroscopy was employed to characterize the chemical composition of Mix/ESM, Bottom/ESM, and Top/ESM. As shown in Figure 1K, all three membrane variants exhibited characteristic protein absorption bands [26], primarily including amide A, B, I, II, and III bands. This spectral profile confirms that the fibrous components of eggshell membranes are predominantly proteinaceous in nature [27], supporting the study’s objective of utilizing ESM-derived fibers as a collagen mimetic reinforcement phase for biomedical applications. Consistent with previous reports on eggshell membrane composition [28,29], the spectra revealed: a broad strong absorption at approximately 3300 cm^−1^, corresponding to the amide A band (N–H stretching vibrations); a weak absorption at 2933 cm^−1^, attributed to the amide B band (C–N stretching vibrations); a prominent peak at 1640 cm^−1^, assigned to the amide I band (C=O stretching vibrations of the peptide backbone), which is widely used for analyzing protein secondary structure; a distinct peak at 1521 cm^−1^, corresponding to the amide II band (N–H bending coupled with C–N stretching); and a weak absorption at 1240 cm^−1^, representing the amide III band (also arising from N–H bending and C–N stretching vibrations) [30]. Notably, comparative analysis of the three membrane types revealed nearly identical peak positions and intensities, indicating highly similar chemical compositions. This compositional consistency demonstrates that the anatomical origin of the eggshell membrane—whether from mixed, bottom, or top portions—does not substantially influence its protein-based chemical structure. Consequently, the selection of ESM fibers for hydrogel reinforcement can be based primarily on morphological and mechanical considerations rather than compositional differences, as all three variants offer comparable protein chemistry suitable for mimicking extracellular matrix architecture.

#### 2.1.3. Mechanical Properties of ESM

The mechanical properties of the three eggshell membrane variants were systematically evaluated to assess their suitability as reinforcing materials. As shown in Figure 1L, the mechanical behavior of Mix/ESM closely resembled that of Top/ESM (outer shell membrane), with both exhibiting substantially higher strength compared to Bottom/ESM (inner shell membrane). Quantitative analysis of tensile fracture strength (Figure 1M) revealed that Bottom/ESM exhibited a 65.52% reduction in strength relative to Mix/ESM (*p* < 0.05), while Top/ESM showed a 25.88% decrease compared to Mix/ESM. In terms of Young’s modulus, Bottom/ESM demonstrated a 15.25% reduction relative to Mix/ESM, whereas Top/ESM displayed a 15.35% increase. Elongation at break analysis (Figure 1N,O) indicated that Bottom/ESM exhibited 58.35% lower elongation than Mix/ESM, and Top/ESM showed a 26.43% reduction, with both membranes demonstrating statistically significant differences from Mix/ESM. These results clearly establish that Mix/ESM possesses superior and well-balanced mechanical properties among the three membrane types. This mechanical advantage can be attributed to its intermediate thickness and composite-like structure, which effectively integrates the mechanical characteristics of both Bottom/ESM and Top/ESM, thereby combining the dense cross-linking of the inner membrane with the robust fiber architecture of the outer membrane. Based on this comprehensive comparative analysis, Mix/ESM emerges as the most suitable candidate for application as a reinforcing material in hydrogel composites, offering an optimal combination of tensile strength, stiffness, and ductility necessary for effective mechanical reinforcement.

### 2.2. Characterization and Properties of ESM/CCS Composites

#### 2.2.1. Morphology of ESM/CCS Composites

**Figure 2:**
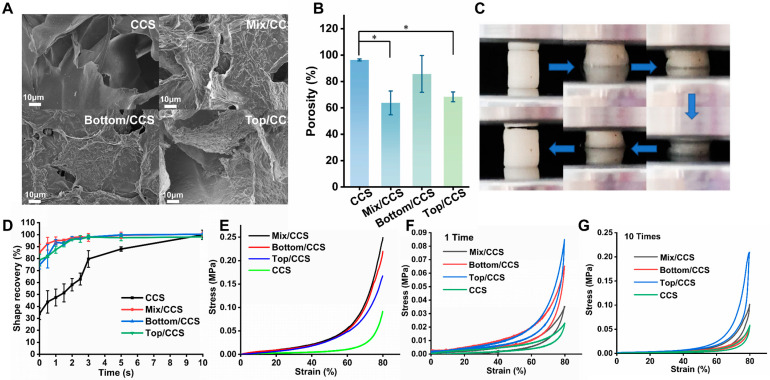
Characterization and properties of ESM/CCS composites. (**A**) The scanning electron microscope images of CCS, Mix/CCS, Bottom/CCS, and Top/CCS hydrogels; (**B**) The porosity statistics of CCS, Mix/CCS, Bottom/CCS, and Top/CCS hydrogels. * *p* < 0.05 (*n* = 3); (**C**) Actual image of the compression process; (**D**) Water-swelling shape memory performance of CCS, Mix/CCS, Bottom/CCS, and Top/CCS hydrogels (*n* = 3); (**E**) Stress–strain curve of single compression; (**F**) Stress–strain curve after one cycle of compression; (**G**) Stress–strain curve after ten cycles of compression.

The microstructural morphology of freeze-dried CCS, Mix/CCS, Bottom/CCS, and Top/CCS hydrogels was examined using scanning electron microscopy (SEM). As shown in Figure 2A, all four hydrogel formulations formed well-defined scaffolds characterized by uniform, interconnected porous networks. Importantly, ESM short fibers derived from Mix/ESM, Top/ESM, and Bottom/ESM were observed to be uniformly adsorbed onto the pore walls of their respective composite hydrogels, confirming successful dispersion and integration of the reinforcing fibers within the chitosan matrix. This homogeneous distribution within the porous architecture is critical for achieving effective load transfer and consistent reinforcement throughout the material. Quantitative porosity analysis using the mass–volume method revealed distinct differences among the four hydrogel systems (Figure 2B). Compared to pristine CCS hydrogel, Mix/CCS exhibited a 33.81% reduction in porosity, representing a statistically significant decrease. Top/CCS showed a similarly substantial reduction of 29.04%, while Bottom/CCS demonstrated a more modest decrease of 11.04%. These variations in porosity can be primarily attributed to differences in fiber length among the three ESM short fiber variants. SEM observations confirmed comparable distribution uniformity across all composites, indicating that dispersion quality was not a differentiating factor. Rather, shorter fibers occupy less volumetric space within the hydrogel pores, resulting in higher retained porosity. The measured fiber length hierarchy—Bottom/ESM > Top/ESM > Mix/ESM—correlates directly with the observed porosity trend—CCS > Bottom/CCS > Top/CCS > Mix/CCS—confirming that fiber length serves as the primary determinant of composite porosity. Notably, despite the porosity reduction upon fiber incorporation, all ESM-reinforced composites-maintained porosity levels well within the range suitable for hemostatic applications. This retained high porosity is essential for facilitating rapid blood fluid absorption and enabling sufficient contact between blood cells and the bioactive chitosan matrix, both of which are critical for effective clot formation. These findings demonstrate that ESM fiber-reinforced chitosan hydrogels combine structural integrity with maintained porosity, rendering them highly suitable for advanced hemostatic dressing applications where both mechanical performance and rapid hemostatic activity are required [31].

#### 2.2.2. Compression Properties of ESM/CCS Composites

The compressive mechanical properties of CCS, Mix/CCS, Bottom/CCS, and Top/CCS hydrogels were systematically evaluated, as shown in Figure 2. Under 80% compressive strain, all four hydrogel formulations exhibited distinct stress responses, with Mix/CCS demonstrating the highest compressive stress—representing a 63% increase compared to pristine CCS hydrogel (Figure 2E). Bottom/CCS and Top/CCS also showed substantial improvements, with stress increases of 58% and 45%, respectively, relative to CCS controls. This marked enhancement in compressive strength can be attributed to the incorporation of eggshell membrane short fibers, which densify the hydrogel’s internal architecture, minimize structural defects, and optimize stress distribution throughout the composite network, thereby effectively improving mechanical performance under load [32]. Following compression to 80% strain, all four hydrogels exhibited excellent shape recovery upon load removal (Figure 2F). Furthermore, after ten consecutive compression cycles, all formulations maintained robust elastic recovery with minimal performance degradation (Figure 2G), confirming their superior compressive elasticity and fatigue resistance. Previous studies have established that interconnected porous structures facilitate uniform load distribution [33], inhibit crack propagation [34], and mitigate permanent deformation under high strain conditions, collectively endowing hydrogels with exceptional compressive resilience [35]. Notably, across both single-cycle and multi-cycle compression testing, Mix/CCS, Bottom/CCS, and Top/CCS consistently outperformed pristine CCS in terms of compressive elasticity and recovery behavior (Figure 2E–G). This outstanding compressive elasticity is of relevance for hemostatic applications. The ability of ESM/CCS composite hydrogels to undergo substantial deformation and rapidly recover enables them to conform tightly to complex wound geometries under applied pressure, ensuring effective contact with bleeding tissues and maintaining consistent pressure delivery throughout the hemostatic process [36]. These mechanical characteristics directly contribute to the superior hemostatic performance observed with ESM-reinforced formulations, establishing a clear structure-function relationship between enhanced compressibility and improved wound sealing capability.

#### 2.2.3. Water-Triggered Shape Memory of ESM/CCS Composites

The water-triggered shape memory performance of CCS, Mix/CCS, Bottom/CCS, and Top/CCS hydrogels is illustrated in Figure 2D. The incorporation of ESM short fibers significantly modulates the hydrogel’s porous architecture and mechanical characteristics, resulting in pronounced differences in compression recovery behavior. When subjected to 80% compressive strain for 1 min, Mix/CCS, Bottom/CCS, and Top/CCS retained 84.96%, 74.62%, and 78.73% of their original volumes, respectively, whereas pristine CCS retained only 33.87% of its initial volume. This substantial variation in compression retention directly influences the subsequent water absorption and volume restoration kinetics. Upon exposure to water, the shape recovery behavior of the four hydrogel formulations diverged markedly. Experimental results demonstrated that Mix/CCS, Bottom/CCS, and Top/CCS achieved approximately 99.51% volume recovery within just 5 s of water absorption. In contrast, CCS required at least 10 s to reach 99.85% recovery, recovering to only 87.9% of its original volume within the first 5 s. These observations conclusively demonstrate the superior water-triggered shape memory performance of ESM-modified hydrogels compared to pure CCS, with fiber-reinforced formulations exhibiting both higher retained volume after compression and accelerated recovery kinetics upon rehydration. The enhanced shape memory characteristics of ESM/CCS composite hydrogels confer significant advantages for hemostatic applications. When dehydrated under compression—a common precondition for storage and deployment—ESM/CCS hydrogels maintain substantially larger residual volumes than CCS, providing more effective physical support for wound compression upon initial placement. Furthermore, their faster recovery kinetics and more robust shape memory properties enable more rapid blood fluid absorption and more efficient adaptation to wound geometries, thereby promoting accelerated hemostasis. These combined attributes render ESM/CCS composite hydrogels particularly well-suited for emergency medical applications where rapid deployment and effective hemorrhage control are critical.

#### 2.2.4. In Vitro Blood Clotting Performance and Hemocompatibility of ESM/CCS Composites

**Figure 3:**
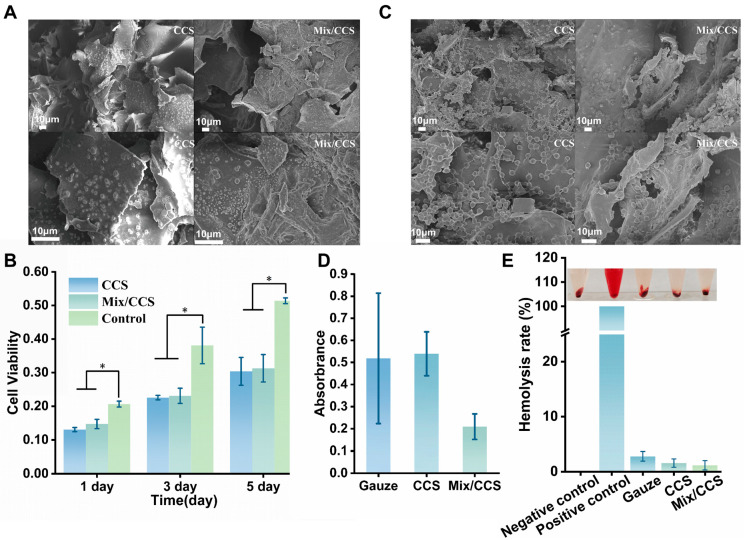
In vitro hemolytic activity and the biological assays of the CCS and Mix/CCS hydrogel. (**A**) SEM image of L929 cells on the surface of the hydrogel after 3 days of culture; (**B**) Bar graph of absorbance values of cells on days 1, 3, and 5. * *p* < 0.05 (*n* = 3); (**C**) SEM image showing the adhesion of red blood cells on the hydrogel; (**D**) Statistical analysis of coagulation supernatant absorbance (*n* = 3); (**E**) Statistical analysis of hemolysis rates and photographs of hemolysis for negative control, positive control, gauze, CCS, and Mix/CCS (*n* = 3).

Based on comprehensive mechanical and structural evaluations, the Mix/CCS hydrogel exhibited optimal overall performance among the tested formulations. Coupled with the practical advantage of Mix/ESM’s ease of acquisition and consistent quality, this formulation was selected for comparative bioactivity characterization against pristine CCS hydrogel in subsequent biological assays. Hemolysis and coagulation tests were conducted to assess the blood compatibility of both hydrogel systems (Figure 3B–E). Visual inspection of hemolysis assay results revealed negligible hemolytic activity for both CCS and Mix/CCS compared to positive controls, with clear supernatant indicative of minimal red blood cell lysis (Figure 3E). Quantitative analysis demonstrated hemolysis rates of 2.797% for gauze, 1.574% for CCS, and 1.175% for Mix/CCS (Figure 3E). Notably, all tested materials exhibited hemolysis rates below 3%, substantially lower than the 5% safety threshold established for blood-contacting biomaterials [37]. Mix/CCS showed a 25.31% reduction in hemolysis compared to CCS, and a 57.91% reduction relative to conventional gauze. These results indicate that incorporation of eggshell membrane microfibers enhances the hemocompatibility of the chitosan matrix, likely attributable to the intrinsic biocompatibility of ESM protein components and the more stable composite network structure. The exceptionally low hemolysis rate of Mix/CCS (<1.2%) confirms its suitability as a blood-contacting hemostatic material.

Coagulation performance was evaluated by measuring supernatant absorbance at 544 nm following 30 min incubation of hydrogels with mouse blood in the presence of 0.2 M CaCl_2_ (Figure 3D). The significantly lower absorbance observed for Mix/CCS compared to CCS and gauze controls indicates superior coagulation activity, as reduced hemoglobin concentration in the supernatant correlates with enhanced clot formation and erythrocyte entrapment within the hydrogel matrix. Quantitative analysis revealed approximately 60% improvement in coagulation performance for Mix/CCS relative to conventional gauze, demonstrating the synergistic effect of chitosan’s intrinsic hemostatic activity and ESM fiber-mediated mechanical reinforcement on blood clot formation. Scanning electron microscopy examination of red blood cell adhesion on hydrogel surfaces (Figure 3C) revealed extensive erythrocyte aggregation within the porous network of Mix/CCS, with visibly enhanced cellular adhesion and clot formation compared to CCS and gauze controls. The observed erythrocyte entrapment and activation within the three-dimensional porous architecture provide structural confirmation of the hydrogel’s coagulation-promoting mechanism, wherein the combination of chitosan’s cationic charge and ESM’s collagen mimetic fiber network facilitates rapid platelet adhesion and fibrin mesh formation. Collectively, these findings establish Mix/CCS as an effective hemostatic dressing characterized by: (1) excellent blood compatibility with hemolysis rate below 1.2%, well within clinically acceptable limits; (2) superior coagulation performance with approximately 60% improvement over conventional gauze; (3) demonstrated capacity for red blood cell aggregation and clot formation; and (4) a safety profile suitable for potential clinical translation. The integration of mechanically reinforcing ESM fibers within the bioactive chitosan matrix thus yields a composite hemostatic material that synergistically combines structural integrity with enhanced biological performance.

#### 2.2.5. Cytocompatibility of ESM/CCS Composites

The cytocompatibility of CCS and Mix/CCS hydrogels was evaluated by directly culturing L929 mouse fibroblasts on the material surfaces, with results presented in Figure 3. Both hydrogel formulations exhibited statistically significant differences in cell viability compared to the control group after 1, 3, and 5 days of co-culture. Notably, cell metabolic activity on both hydrogels increased progressively from day 1 to day 5 (Figure 3B), indicating sustained cell proliferation and confirming the low cytotoxicity of both CCS and Mix/CCS matrices. Morphological examination by SEM after three days of culture (Figure 3A) revealed abundant cell adhesion and spreading on the porous architecture of both hydrogels, with cells exhibiting typical fibroblast morphology and establishing intimate contact with the scaffold surfaces. Quantitative assessment using the CCK-8 assay demonstrated that the Mix/CCS group exhibited 12.63%, 2.36%, and 3.02% higher absorbance values compared to the CCS group on days 1, 3, and 5, respectively (Figure 3B). This consistently enhanced metabolic activity across all time points, particularly the pronounced improvement at early culture stages, suggests that Mix/CCS provides a more favorable microenvironment for initial cell attachment and subsequent proliferation. The improved cytocompatibility of Mix/CCS can be attributed to the incorporation of eggshell membrane microfibers, which structurally mimic native collagen fiber architecture and provide biochemical cues that enhance cell–scaffold interactions [38]. The collagen mimetic protein composition of ESM fibers, including type I collagen and fibronectin, likely promotes integrin-mediated cell adhesion and facilitates extracellular matrix deposition, thereby supporting more robust cell growth compared to pure chitosan hydrogels [39]. Taken together, these results demonstrate that Mix/CCS hydrogel exhibits superior cytocompatibility relative to unmodified CCS, supporting enhanced cell adhesion, spreading, and proliferation throughout the culture period. This favorable biological response, combined with the previously established mechanical and hemostatic advantages, positions Mix/CCS as a highly suitable candidate for biocompatible hemostatic dressing applications where both material performance and tissue compatibility are critical requirements.

#### 2.2.6. In Vivo Hemostatic Capacity of ESM/CCS Composites

**Figure 4:**
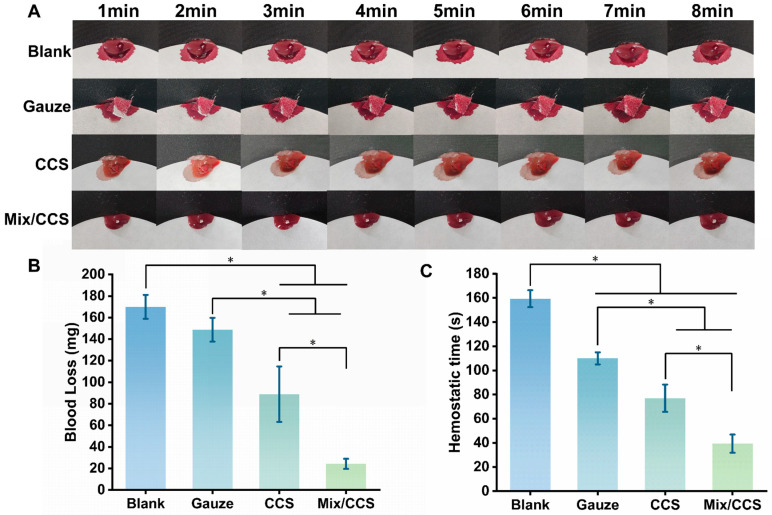
The hemostatic performance of the CCS and Mix/CCS hydrogel in the mouse liver trauma model. (**A**) Representative images of blood loss in the blank group, gauze group, CCS group, and Mix/CCS group; (**B**) Bar chart showing the amount of blood loss. * *p* < 0.05 (*n* = 3); (**C**) Bar chart showing hemostasis time. * *p* < 0.05 (*n* = 3).

The in vivo hemostatic performance of Mix/CCS hydrogel was evaluated using a mouse liver injury model to simulate deep internal organ hemorrhage, with results presented in Figure 4.

Total blood loss differed substantially across experimental groups (Figure 4B): the blank control group exhibited the highest blood loss at 169.97 ± 11.09 mg, followed by the gauze group at 148.73 ± 11.05 mg, the CCS hydrogel group at 88.93 ± 25.76 mg, and the Mix/CCS hydrogel group at 24.27 ± 4.70 mg. Application of Mix/CCS hydrogel resulted in dramatically reduced hemorrhage compared to all control groups (*p* < 0.05), corresponding to an 85.72% reduction in blood loss relative to the blank group and a 72.71% reduction relative to the CCS hydrogel group (Figure 4A). The Mix/CCS hydrogel demonstrated a similarly superior performance in hemostasis time (Figure 4C). Mean hemostasis times for the blank, gauze, CCS, and Mix/CCS groups were 159.33 ± 7.02 s, 110 ± 5 s, 77 ± 11.27 s, and 39.33 ± 7.57 s, respectively. The hemostasis time achieved with Mix/CCS was significantly shorter than that of all control groups (*p* < 0.05), representing a 75.32% reduction compared to the blank group and a 48.92% reduction compared to the CCS hydrogel group. These results unequivocally demonstrate that Mix/CCS hydrogel substantially outperforms both conventional gauze and unmodified CCS hydrogel in controlling severe liver hemorrhage. The enhanced hemostatic efficacy of Mix/CCS can be attributed to several synergistic mechanisms arising from ESM fiber incorporation. First, the reduced porosity of the composite hydrogel (previously documented in Figure 2B) results in greater volumetric expansion upon blood absorption, enabling earlier and more effective wound contact to initiate hemostasis. Second, the superior shape memory properties of Mix/CCS (demonstrated in Figure 2D) accelerate blood fluid absorption and expedite volumetric recovery, thereby promoting rapid sealing of the wound site [40]. Third, the maintained porous architecture, combined with the bioactive chitosan matrix and collagen mimetic ESM fibers, provides an optimized scaffold for erythrocyte aggregation and platelet activation, further enhancing clot formation kinetics. Collectively, these findings establish that the incorporation of eggshell membrane short fibers significantly enhances the hemostatic capability of chitosan-based hydrogels, with Mix/CCS achieving rapid hemorrhage control in a challenging internal organ injury model. The combination of substantially reduced blood loss and markedly shortened hemostasis time positions Mix/CCS as a highly promising hemostatic dressing for clinical applications requiring rapid and effective control of deep, non-compressible bleeding.

## 3. Conclusions

In this study, we successfully developed a novel composite hydrogel (Mix/CCS) by integrating uniform eggshell membrane (ESM)-derived nanofibrils into a citric-acid-crosslinked chitosan matrix. The resulting material exhibited an optimized porous architecture (63.78% porosity) with homogeneously dispersed ESM fibers along pore walls, effectively overcoming the dispersion challenges that have historically limited continuous nanofiber reinforcement strategies. This structural optimization translated directly into exceptional mechanical performance: Mix/CCS demonstrated 63% higher compressive strength than pristine CCS hydrogel, attributed to effective load transfer between the chitosan matrix and collagen mimetic ESM fibers, as well as superior shape memory with complete volume recovery within 5 s of water absorption—twice the rate of unmodified CCS. Blood compatibility assessment revealed a hemolysis rate of only 1.175% (25.31% lower than CCS) and 61.03% lower coagulation supernatant absorbance than CCS, indicating markedly enhanced clot formation capacity through a synergistic combination of chitosan’s erythrocyte-binding properties and ESM’s collagen mimetic platelet adhesion sites. Cytotoxicity evaluation confirmed excellent cytocompatibility, with Mix/CCS supporting enhanced L929 fibroblast adhesion and proliferation due to bioactive protein sequences (type I collagen, fibronectin) within ESM fibers. In vivo validation using a mouse liver injury model demonstrated rapid hemostasis in approximately 39 s, reducing blood loss by 85.72% versus untreated controls and 72.71% versus CCS. This work introduces three synergistic innovations: (1) utilizing ESM nanofibrils as a renewable, collagen mimetic reinforcing phase combining superior dispersibility with preserved bioactivity; (2) achieving multifunctionality through triple synergy of chitosan bioactivity, ESM’s ECM-like architecture, and non-toxic citric acid crosslinking; and (3) valorizing abundant biological waste into high-performance biomedical materials. While future studies in larger animal models and investigation of long-term degradation behavior would strengthen translational confidence, the Mix/CCS hydrogel integrates enhanced mechanical strength, rapid shape recovery, excellent blood compatibility, and proven in vivo hemostatic efficacy into a single sustainable platform, representing a promising advanced hemostatic dressing for complex wound management and establishing a broader biomimetic paradigm for engineering functional materials from naturally occurring nanofiber architectures.

## 4. Materials and Methods

### 4.1. Materials

Chitosan (Shanghai Mai Rui Er Chemical Technology Co., Ltd., Shanghai, China); Acetic acid (glacial acetic acid, molecular weight 60.05, Nanjing Chemical Reagent Co., Ltd., Nanjing, China); Citric acid (molecular weight 210.14, Jiangsu Rugao Chemical Reagent Factory, Rugao, China); Phosphate-buffered saline (PBS, Shanghai Yuan Ye Biotechnology Co., Ltd., Shanghai, China); Trypsin (molecular weight 24,000, Tianjin Lvyang Biological Products Technology Co., Ltd., Tianjin, China); Dulbecco’s Modified Eagle Medium (DMEM, HyClone, Logan, UT, USA); Fetal bovine serum (molecular weight 66,430, Zhejiang Tianhang Bio-Tech Co., Ltd., Huzhou, China); Penicillin-streptomycin solution (molecular weight 915.67, Tianjin Lvyang Biological Products Technology Co., Ltd., Tianjin, China); CCK-8 (Feiyu Bio, Nantong, China); Cell activity and cytotoxicity assay kit (Beyotime, Shanghai, China); Glutaraldehyde 25% aqueous solution (molecular weight 100.12, Ron Reagent, Shanghai, China);Sodium chloride (NaCl, molecular weight 58.44, Xi Long Chemical Co., Ltd., Shantou, China) and sodium hydroxide (NaOH, molecular weight 40, Xi Long Chemical Co., Ltd., Shantou, China).

### 4.2. Preparation

#### 4.2.1. Natural Eggshell Membrane Short Fiber

Collect discarded eggshells and soak them in clean water for a period of 2–4 h. After the shells and membranes are wetted, manually peel off the eggshell membranes (Mix/ESM). Cut the eggshell and membrane along the air cell’s midpoint from the blunt end and separate the membrane from the air cell. The membrane on the side facing the egg white is the inner eggshell membrane (Bottom/ESM), while the membrane on the side facing the eggshell is the outer eggshell membrane (Top/ESM). After obtaining Mix/ESM, Bottom/ESM, and Top/ESM, clean them with clean water, remove surface moisture, and cut them into small pieces. Place the pieces into a mortar and add liquid nitrogen to fully submerge them. During the evaporation of the liquid nitrogen, continue grinding and repeat this process three times. Dry the resulting crude product and continue grinding for 20–30 min until the powder shows no obvious static electricity, thus obtaining eggshell membrane short fibers.

#### 4.2.2. Citric Acid Modified Chitosan Hydrogel (CCS)

Chitosan (0.21 g) was dispersed in a 3% wt acetic acid aqueous solution and stirred at room temperature overnight. Once fully dissolved, 0.09 g of citric acid was added, and the mixture was stirred for 6 h at room temperature until dissolved, with air bubbles removed by standing. The sample solution was then slowly poured into a 24-well plate mold and placed in a −20 °C freezer for 24 to 30 h to freeze and solidify. After removal from the freezer, an appropriate amount of 0.5 mol/L NaOH solution was added to the sample surface and soaked for 1 h. The sample was then fully thawed for over 3 h and rinsed thoroughly with distilled water until neutral, yielding the citric-acid-modified chitosan hydrogel (CCS).

#### 4.2.3. Natural Nanofiber-Based Citric-Acid-Modified Chitosan Hydrogel (ESM/CCS)

Chitosan (0.21 g) was dispersed in a 3% wt acetic acid aqueous solution and stirred at room temperature overnight. Once fully dissolved, 0.3 g of eggshell membrane short fibers and 0.09 g of citric acid were added, and the mixture was stirred at room temperature for 6 h until dissolved, with air bubbles removed by standing. The sample solution was then slowly poured into a mold and placed in a −20 °C freezer to freeze and solidify for 24 to 30 h. After removal from the freezer, an appropriate amount of 0.5 mol/L NaOH solution was added to the surface of the sample and soaked for 1 h. The sample was then fully thawed for over 3 h and rinsed thoroughly with distilled water until neutral, yielding the eggshell membrane-based citric-acid-modified chitosan hydrogel (Mix/CCS), outer eggshell membrane-based citric-acid-modified chitosan hydrogel (Top/CCS), and inner eggshell membrane-based citric-acid-modified chitosan hydrogel (Bottom/CCS).

### 4.3. Characterization of Natural Eggshell Membrane Fibers (ESM)

#### 4.3.1. Morphology Observation of ESM

The prepared eggshell membrane and the eggshell membrane short fibers were dried, and their surface topography was observed using a field emission scanning electron microscope (FE-SEM, ZEISS Gemini SEM 300, ZEISS, Oberkochen, Germany). The fiber diameter and length were measured using the ImageJ software (1.54j), and the average diameter and length were calculated. The number of samples used was more than 50.

#### 4.3.2. Infrared Spectrum Analysis of ESM

The Fourier-transform infrared spectrometer (FT-IR, TENS0R 27, Bruker Corporation, Billerica, MA, USA) was used at room temperature to obtain the infrared spectra of Mix/ESM, Top/ESM, and Bottom/ESM. The FT-IR spectra were utilized to confirm and compare the compositions of the three types of eggshell membranes.

#### 4.3.3. Mechanical Property Test of ESM

The mechanical properties of eggshell membrane were tested by electric tensile testing machine. The setting parameters of the tensile instrument are: the distance between the two jaws is 20 mm, the tensile rate is 1 mm/s, the range of the force sensor is 10 N, and the stress–strain curve is obtained. All tests were carried out at a temperature of 18~25 °C and a relative humidity of 40~60%.

### 4.4. Characterization of Citric Acid Modified Chitosan Hydrogel with Natural Nano-Fibers (ESM/CCS)

#### 4.4.1. Morphology Observation of ESM/CCS

The prepared hydrogels were frozen at −80 °C and freeze-dried with a freeze dryer (FD-1C-50, Shanghai Bilang Instrument Manufacturing Co., Ltd., Shanghai, China) to prepare dry hydrogels. The surface morphology of dry hydrogels was observed by scanning electron microscope (SEM) and photographed. Find the weight (W1) and volume (V) of three groups of hydrogels in the wet state after absorbing anhydrous ethanol and the weight (W0) of the dry hydrogel after freeze-drying, and calculate the porosity of different hydrogel systems according to the formula by using the density (ρ) of anhydrous ethanol. The porosity of different hydrogel systems (P) was calculated as follows:

P = ((W1 − W0)/ρV) × 100%(1)

#### 4.4.2. Compression Performance Test of ESM/CCS

The compressive properties of CCS, Mix/CCS, Top/CCS and Bottom/CCS hydrogels at room temperature were evaluated using a tensile testing machine. The cylindrical hydrogels with a diameter of 7 mm and a height of 8 mm were prepared. The maximum compression strain is 80% and the compression rate is 0.1 mm/s. After the frozen hydrogel absorbs water, the compressive strain is first applied to a preset strain, then released to 0% strain, and it is circulated for 10 times.

#### 4.4.3. Shape-Memory Behavior of ESM/CCS

A cylindrical hydrogel sample with an initial height *h* was first allowed to reach swelling equilibrium in distilled water. Subsequently, the hydrogel was compressed to 80% strain and maintained at this strain for 1 min, during which water squeezed out from the hydrogel was absorbed by filter paper. The dehydrated hydrogel was then re-immersed in water, and the height recovery of the hydrogel was recorded as a function of time.

#### 4.4.4. Hemolytic Activity Assay of ESM/CCS

Freeze-dried hydrogel (10 mg) was weighed respectively, and normal saline (4.5 mL) and sodium-citrate-stabilized rat blood (200 μL) were added. The above solution was incubated at 37 °C for 1 h and then centrifuged at 3000 rpm for 10 min to obtain the supernatant. The absorbance of the supernatant was measured at 540 nm using a multi-labeled microplate detector (Enspire 2300, PerkinElmer, Shelton, CT, USA). HP was calculated using the following formula:

HP (%) = [(Dt − Dpc)/(Dnc − Dpc)] × 100%(2)

where Dt, Dpc and Dnc represent the absorbance values of the material, negative control (physiological saline), and positive control (0.1%Triton X-100) groups, respectively. Each group consisted of three replicates.

#### 4.4.5. Coagulation Test of ESM/CCS

In total, 5 mg of freeze-dried hydrogel were weighed respectively; 200 μL of sodium-citrate-stabilized rat blood and 10 μL of CaCl_2_ (0.2 m) were slowly added, followed by shaking at 37 °C for 30 min. After washing with distilled water three times, the suspension was collected, and the absorbance was measured at 544 nm using an ultraviolet spectrophotometer. The gauze group was used as a blank control, and each group contained three replicates.

#### 4.4.6. Cell Cytotoxicity of ESM/CCS

The cultured L929 cells were taken out of the incubator, immediately transferred to a centrifuge tube, and added to 3 mL of basic medium (DMEM+10% FBS+1% penicillin-streptomycin) for centrifugation; the rotation speed was set at 1000 r/min for 3 min. Then, the supernatant was removed, and 2 mL of basic culture solution was added to suspend the cells. After 2 days of culture in a culture bottle at a ratio of 1:5, the cells were separated with 0.125% trypsin -EDTA solution and counted with a blood cell counting plate and an optical microscope. Hydrogels of different systems were sterilized by a high-pressure steam pot at 121 °C for 15 min, and then sterilized under ultraviolet light for 30 min. After that, hydrogels of different systems were placed in 24-well plates, and 10,000 cells per well were seeded for 1, 3 and 5 days. After each period of culture, it was fixed with 4% glutaraldehyde overnight and then frozen at −80 °C for one night and then dried in a vacuum drying instrument for one day. The dried hydrogel was quenched in liquid nitrogen, observed by SEM and photographed.

The revived L929 cells were planted on hydrogels of different systems, and the culture plate was used as a blank control group—cultured for 1, 3 and 5 days, respectively. After each period of culture, cck8 was added and incubated in an incubator for two hours. Finally, 100 μL of each well was sucked into a 96-well plate by a pipetting gun according to the corresponding sequence, and the absorption value was detected by an enzyme-labeled instrument at 450 nm and the corresponding data was recorded.

#### 4.4.7. In Vivo Hemostatic Capacity of ESM/CCS

The hemostatic ability of the hydrogel was evaluated by the mouse liver trauma model. Male mice (6 weeks old, 25–32 g) were obtained from the animal center of Nantong University and randomly divided into four groups. All animal experiments complied with the guidelines of the National Research Council’s Guide for the Care and Use of Laboratory Animals and were approved by the Animal Ethics Committee of Nantong University (China). Before hemostasis, the hydrogel was cut into cylindrical shapes (3 mm in diameter) and compressed into a contracted shape by squeezing water. After anesthesia, the mouse liver was exposed through an abdominal incision, and the slurry around the liver was carefully removed. A cylindrical wound (3 mm in diameter) was made on the mouse liver as an incompressible wound model, and gauze, CCS, and Mix/CCS hydrogel were immediately applied to the bleeding site. Untreated wounds were used as a blank control group, with 3 rats in each group.

### 4.5. Statistical Analysis

In order to test whether there is a significant difference between samples, we used Origin 8.0 statistical software package to analyze the data. For the significant difference, we used one-way ANOVA. The difference between the two groups was tested by a *t*-test. Specifically, when *p* < 0.05, it was considered that there was a significant difference (*).

## Data Availability

The data supporting the findings of this study are available from the corresponding author upon reasonable request.

## Abbreviations

The following abbreviations are used in this manuscript:

ESM	Eggshell membrane
ECM	Extracellular matrix
CCS	Citric-acid-crosslinked chitosan
FTIR	Fourier transform infrared
SEM	Scanning electron microscopy
